# The glycation level of milk protein strongly modulates post-prandial lysine availability in humans

**DOI:** 10.1017/S0007114519002927

**Published:** 2019-11-15

**Authors:** Jean Nyakayiru, Glenn A. A. van Lieshout, Jorn Trommelen, Janneau van Kranenburg, Lex B. Verdijk, Marjolijn C. E. Bragt, Luc J. C. van Loon

**Affiliations:** 1 Department of Human Biology, NUTRIM School of Nutrition and Translational Research in Metabolism, Maastricht University Medical Centre+, 6229 ER Maastricht, the Netherlands; 2 FrieslandCampina, 3818 LE Amersfoort, the Netherlands

**Keywords:** Whey, Casein, Protein powder, Infant formula, Maillard reactions

## Abstract

Industrial heat treatment of milk results in protein glycation. A high protein glycation level has been suggested to compromise the post-prandial rise in plasma amino acid availability following protein ingestion. In the present study, we assessed the impact of glycation level of milk protein on post-prandial plasma amino acid responses in humans. Fifteen healthy, young men (age 26 (SEM 1) years, BMI 24 (SEM 1) kg/m^2^) participated in this randomised cross-over study and ingested milk protein powder with protein glycation levels of 3, 20 and 50 % blocked lysine. On each trial day, arterialised blood samples were collected at regular intervals during a 6-h post-prandial period to assess plasma amino acid concentrations using ultra-performance liquid chromatography. Plasma essential amino acid (EAA) concentrations increased following milk protein ingestion, with the 20 and 50 % glycated milk proteins showing lower overall EAA responses compared with the 3 % glycated milk protein (161 (SEM 7) and 142 (SEM 7) *v*. 178 (SEM 9) mmol/l × 6 h, respectively; *P* ≤ 0·011). The lower post-prandial plasma amino acid responses were fully attributed to an attenuated post-prandial rise in circulating plasma lysine concentrations. Plasma lysine responses (incremental AUC) following ingestion of the 20 and 50 % glycated milk proteins were 35 (SEM 4) and 92 (SEM 2) % lower compared with the 3 % glycated milk protein (21·3 (SEM 1·4) and 2·8 (SEM 0·7) *v*. 33·3 (SEM 1·7) mmol/l × 6 h, respectively; *P* < 0·001). Milk protein glycation lowers post-prandial plasma lysine availability in humans. The lower post-prandial availability of lysine following ingestion of proteins with a high glycation level may compromise the anabolic properties of a protein source.

Ingestion of dietary protein directly stimulates muscle protein synthesis rates^(^^1^^–^^3^^)^. This anabolic response is regulated at various levels, starting from protein digestion, the absorption of free amino acids in the gastrointestinal tract, their partial release into the systemic circulation, the uptake of amino acids in skeletal muscle tissue, changes in intramyocellular signalling and eventually incorporation of amino acids into muscle proteins, that is, muscle protein synthesis^(^^4^^,^^5^^)^. Factors such as the amount and the type of dietary protein ingested have been shown to strongly modulate the post-prandial rise in circulating amino acid concentrations and, as such, the post-prandial stimulation of muscle protein synthesis^(^^1^^,^^6^^–^^10^^)^.

Extensive research performed throughout the past two decades has revealed distinct differences in the digestion and absorption kinetics of different milk proteins. Previous work has shown that plasma amino acid responses differ following the ingestion of casein *v.* whey protein^(^^6^^,^^9^^)^, casein *v.* casein hydrolysate^(^^4^^)^ and casein ingested within or separate from the milk matrix^(^^11^^)^. Throughout the literature, substantial differences have been observed in the post-prandial plasma amino acid responses following the ingestion of such proteins or protein fractions^(^^4^^–^^6^^,^^9^^,^^12^^)^. These apparent differences do not seem to be explained merely by subjects’ characteristics or the type of protein provided, but may instead be attributed to the industrial processing procedures applied prior to protein consumption.

Production of milk protein powders requires milk to be exposed to heat through procedures such as roller drying (about 160°C) or spray drying (about 200°C)^(^^13^^,^^14^^)^. In addition to obtaining the desired level of dehydration, drying of milk protein leads to protein glycation resulting from the so-called Maillard reactions^(^^13^^–^^15^^)^. Depending on the heat treatment applied, the Maillard reactions can lead to the formation of early, advanced and late Maillard reaction products^(^^16^^)^. The early Maillard reaction consists of the condensation of a reducing sugar (e.g. lactose) with an amino acid, with the essential amino acid (EAA) lysine being more susceptible to this reaction^(^^17^^–^^19^^)^. Formation of furosine following acid hydrolysis of a dietary protein is often used as a marker of early Maillard reaction products to calculate the level of blocked lysine^(^^20^^)^. Although protein glycation is strongly potentiated by the high temperatures applied during protein processing, several studies have shown that protein glycation also continues to occur during storage at room temperature^(^^13^^,^^21^^,^^22^^)^. Indeed, while there is no protein glycation in fresh raw milk, commercially available milk protein powders such as infant formulas and protein supplements have been reported to show blocked lysine levels as high as 55 %^(^^23^^–^^26^^)^.

An increasing number of studies indicate that milk protein glycation (GLYC) can strongly affect protein digestibility^(^^27^^–^^32^^)^. *In vitro* gastrointestinal digestion studies have shown that high levels of protein glycation impair the effective enzymatic hydrolysis of protein, which may impair subsequent protein-derived amino acid absorption^(^^27^^–^^29^^)^. In accordance, a study in pigs has shown an attenuated rise in circulating amino acid levels following ingestion of glycated *v*. non-glycated milk protein, with lysine availability being particularly compromised^(^^31^^)^. As the increase in protein synthesis following dietary protein intake has been associated with the EAA content of the protein^(^^10^^)^, reduced post-prandial lysine availability due to protein glycation may limit the ability to maximise this process. However, there are currently no data available on whether the level of protein glycation modulates the post-prandial plasma amino acid response in humans.

We hypothesised that a greater protein glycation level would compromise protein digestion and amino acid absorption, resulting in an attenuated post-prandial rise in circulating amino acid concentrations in humans. To test our hypothesis, fifteen young males were recruited to participate in a study in which we assessed the plasma amino acid responses following ingestion of a milk protein powder with a low (3 %), moderate (20 %) and high (50 %) level of protein glycation.

## Methods

### Participants

Fifteen healthy young male subjects were recruited to participate in this randomised cross-over study. Subjects’ characteristics are presented in Table 1. Potential subjects were included if they were non-smoking, recreationally active (exercised ≤3 times per week) and had no history of lactose intolerance. The participants were informed about the experimental procedures and possible risks of participation prior to signing an informed consent. The study was approved by the Medical Ethical Committee of the Maastricht University Medical Centre, The Netherlands, and was registered at the Nederlands Trial Register (NTR6843) (https://www.trialregister.nl/trial/6673). All procedures were carried out in accordance with the standards stated in the 2013 version of the Helsinki Declaration.

**Table 1.** Subjects’ characteristics (*n* 15) (Mean values with their standard errors)

	Mean	sem
Age (years)	26	1
Weight (kg)	81	2
Height (m)	1·82	0·01
BMI (kg/m^2^)	24	1
LBM (kg)	62	2
Body fat (%)	20	1

LBM, lean body mass.

#### Study design

This double-blind randomised cross-over study assessed the post-prandial plasma amino acid response following the ingestion of 40 g milk protein powder with three different levels of protein glycation. During the 3 test days in the present study, subjects ingested the milk protein powder dissolved in water, followed by a 6-h post-prandial assessment period during which multiple blood samples were collected while subjects remained in the seated position. Test days were separated by at least 7 d of wash-out.

#### Pre-testing

Prior to inclusion into the study, subjects first completed a screening session which consisted of assessment of body weight and height, as well as an assessment of body composition by dual-energy X-ray absorptiometry scan (Discovery A; Hologic). All subjects were deemed healthy based on their responses to a routine medical screening questionnaire.

#### Standardisation of physical activity and diet

Participants refrained from strenuous physical activity in the 48 h leading up to the trial days. Physical activity and dietary intake were recorded during 48 h prior to the first experimental test day and were replicated in the 48 h prior to the second and third test day. On the evening prior to each of the 3 test days, all participants consumed a standardised dinner (54 kJ/kg) providing 61 % energy as carbohydrate, 25 % energy as fat and 14 % energy as protein.

### Experimental procedures

**Fig. 1.**
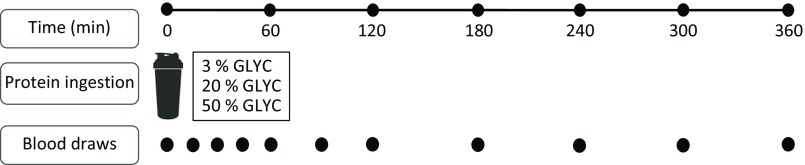
Schematic representation of the study design. GLYC, milk protein glycation level.

Subjects reported to the laboratory by car or public transport at 08.00 hours on trial days following an overnight fast. The experimental protocol during each test day is shown in Fig. 1. Each test day started with placement of a Teflon catheter into a dorsal hand vein for arterialised blood draws. To allow sampling of arterialised blood, the hand was first placed in a hot box (60°C) for 10 min before drawing blood. After collection of a basal blood sample, participants consumed a milk protein beverage within 5 min (*t* = 0 min). Consumption of the milk protein beverage was then followed by a 6-h post-prandial period in which ten blood samples were collected at *t* = 15, 30, 45, 60, 90, 120, 180, 240, 300 and 360 min while subjects remained in the seated position.

### Milk protein intervention

The milk protein powder assessed in the present study had a protein concentration of 42 % (% N × 6·38) and a whey:casein protein ratio of 60:40, a lactose concentration of 39 %, a fat concentration of 9 % and a mineral concentration of 4 % (FrieslandCampina Innovation Centre). A whey:casein protein ratio of 60:40 was chosen to mimic the ratio in infant formula. Spray drying with minimal heat load was performed to obtain a protein powder with 3 % blocked lysine (3 % GLYC). For the medium (20 % GLYC) and high (50 % GLYC) glycated milk protein powder, the 3 % GLYC was incubated in a stove (50°C, *a*_w_ 0·35) to induce the desired protein glycation level. Milk powder with a blocked lysine level of 20 % (20 % GLYC) was obtained by incubation of the 3 % GLYC for 55 h, whereas an incubation for 504 h was required to obtain a milk powder with a blocked lysine level of 50 % (50 % GLYC). The milk powder mixtures were dissolved in water on test days to a total volume of 600 ml. All the interventional milk protein beverages were flavoured with 3 ml vanilla flavour (Dr Oetker).

### Assessment of protein glycation

Lysine blockage was assessed by quantifying lysine and furosine content in the milk protein powders after acid hydrolysis using ion-pair reversed-phase HPLC (Ansynth Service B.V.)^(^^18^^)^. Furosine is formed during acid hydrolysis of protein-bound Amadori products (lactulosyl-lysine, fructosyl-lysine and tagatosyl-lysine)^(^^33^^)^. The fact that furosine does not occur naturally in dairy products makes it a reliable marker of lysine blockage when assessed under controlled conditions^(^^17^^,^^34^^)^. Furosine contents measured after acid hydrolysis of the milk protein powders were 0·58 (SEM 0·01), 4·4 (SEM 0·01) and 11·3 (SEM 0·01) g/kg powder for the 3, 20 and 50 % glycated milk protein, respectively. The furosine contents were subsequently used to calculate the level of lysine blockage following determination of total lysine contents with HPLC. Total lysine content in the milk proteins was 9·7 g/100 g protein. Total reactive lysine contents were 9·5 (SEM 0·02), 7·8 (SEM 0·03) and 4·8 (SEM 0·03) g protein for the 3, 20 and 50 % glycated milk protein, respectively.

### Plasma analysis

Arterialised blood samples were collected in 8 ml EDTA containing tubes and immediately centrifuged at 1000 ***g*** for 10 min, at 4°C. Aliquots of plasma were immediately frozen in liquid N_2_ and stored at −80°C for subsequent analyses. Plasma glucose and insulin concentrations were analysed using commercially available kits (GLUC3, catalogue no. 05168791190, and Immunologic, catalogue no. 12017547122; Roche). Quantification of plasma amino acid concentrations was performed using ultra-performance liquid chromatography MS (ACQUITY UPLC H-Class with QDa; Waters). Plasma (50 µl) was deproteinised using 100 µl of 10 % sulfosalicylic acid with 50 µm of MSK-A2 internal standard (Cambridge Isotope Laboratories). Subsequently, 50 µl of demineralised water was added and samples were centrifuged. After centrifugation, 10 µl of supernatant was added to 70 µl of borate reaction buffer (Waters). In addition, 20 µl of AccQ-Tag derivatising reagent solution (Waters) was added after which the solution was heated to 55°C for 10 min. Of this 100 µl derivative, 1 µl was injected and measured using ultra-performance liquid chromatography MS.

To quantify the possible absorption and subsequent release of blocked lysine in the circulation, we assessed plasma furosine concentrations after acid hydrolysis of deproteinised plasma samples from a subset of the individuals (*n* 2). However, plasma furosine concentrations remained below the detection level of 2 µmol/l, implying that blocked lysine is not substantially absorbed/synthesised *in vivo* in healthy individuals.

### Statistical analysis

A sample size of fifteen subjects, including 15 % dropout, was calculated with a power of 80 % and an *α* of 0·05/3 (for three treatment arms) to detect a relevant difference between plasma lysine values of 53 μmol/l. Potential differences in plasma glucose, insulin and amino acid concentrations were statistically assessed using two-way repeated-measures ANOVA with time and treatment (3 % GLYC, 20 % GLYC and 50 % GLYC) as within-subject factors. Incremental AUC (iAUC) representing the *t* = 0–360 min period was assessed using one-way repeated-measures ANOVA. Observed main effects or interactions were further assessed with Bonferroni-corrected *post hoc* testing where appropriate. Statistical significance was set at *P* < 0·05. All calculations were performed using SPSS Statistics (version 25; IBM) and are presented as mean values with their standard errors.

## Results

### Plasma glucose and insulin concentrations

**Fig. 2.**
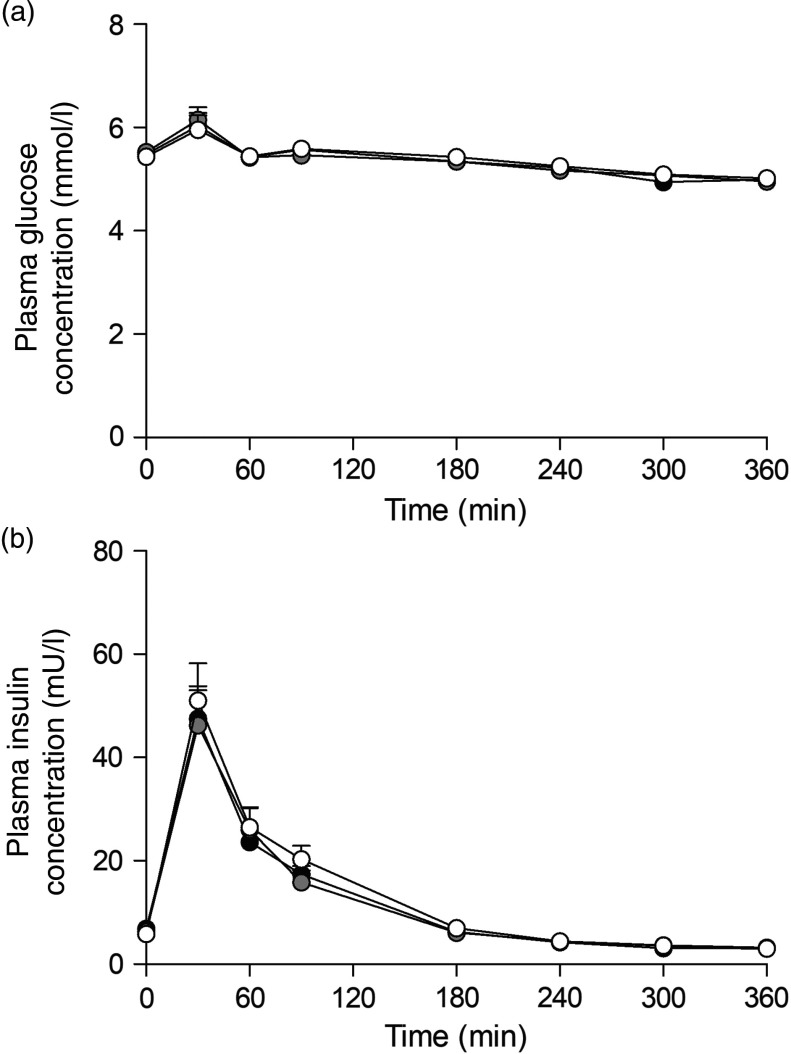
Plasma glucose (a) and insulin (b) concentrations. Values are means with their standard errors (*n* 15). 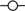, 3 % milk protein glycation (GLYC); 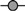, 20 % GLYC; 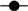, 50 % GLYC.

Ingestion of the three milk protein drinks resulted in a rise in circulating plasma glucose concentrations (Fig. 2(a)), after which levels gradually declined over time (time effect: *P* < 0·001). Plasma insulin concentrations (Fig. 2(b)) increased in a similar fashion, peaking within 60 min after protein ingestion, after which concentrations declined back towards baseline values (time effect: *P* < 0·001). No differences were observed for plasma glucose or insulin concentrations between the three glycated milk protein drinks.

### Plasma amino acids concentrations

**Fig. 3.**
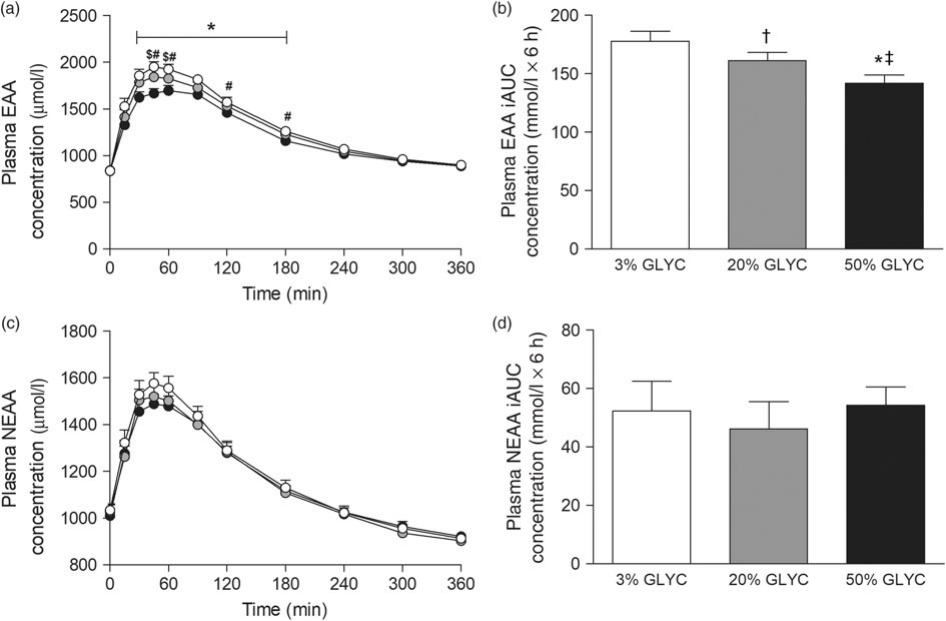
Plasma essential amino acids (EAA; a and b) and non-essential amino acid concentrations (NEAA; c and d). Values are means with their standard errors (*n* 15). 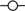, 3 % milk protein glycation (GLYC); 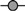, 20 % GLYC; 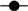, 50 % GLYC. iAUC, incremental AUC. * Significantly lower concentrations following ingestion of 50 % GLYC than 3 % GLYC (*P* ≤ 0·007). † Significantly lower concentrations following ingestion of 20 % GLYC than 3 % GLYC (*P* ≤ 0·021). ‡ Significantly lower concentrations following ingestion of 50 % GLYC than 20 % GLYC (*P* ≤ 0·005).

Plasma EAA and non-essential amino acid (NEAA) concentrations are shown in Fig. 3. Consumption of the milk proteins resulted in a sustained increase in plasma EAA and NEAA concentrations (time effect: *P* < 0·001), but the response was only different between treatments for plasma EAA (time × treatment interaction *P* < 0·001). Plasma EAA concentrations following ingestion of 50 % GLYC were lower from *t* = 30–180 min when compared with 3 % GLYC (*P* ≤ 0·021; Fig. 3(a)). Similarly, ingestion of 20 % GLYC showed lower plasma EAA concentrations when compared with 3 % GLYC from *t* = 45–60 min (*P* ≤ 0·003). A significant difference between treatments (*P* < 0·001) was also observed for the post-prandial plasma EAA response during the entire 6-h period (iAUC), with 20 % GLYC (161 (SEM 7) mmol/l × 6 h) and 50 % GLYC (142 (SEM 7) mmol/l × 6 h) showing 8 (SEM 3) % and 20 (SEM 2) % lower concentrations than 3 % GLYC (178 (SEM 9) mmol/l × 6 h), respectively (*P* ≤ 0·011; Fig. 3(b)). In contrast to the EAA, no between-treatment differences were observed over time in plasma NEAA concentrations (*P* ≥ 0·221; Fig. 3(c) and (d)).

**Fig. 4.**
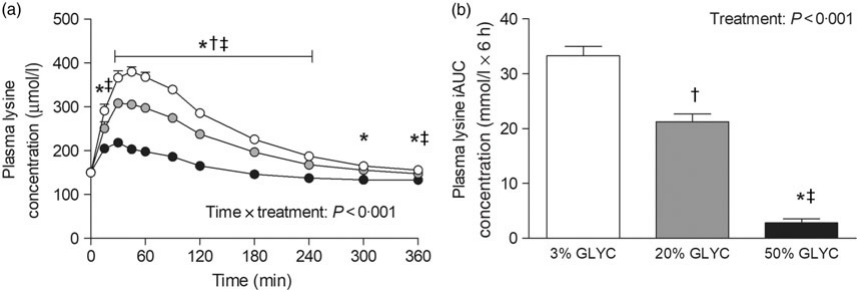
Plasma lysine concentrations (a), and incremental AUC (iAUC; b). Values are means with their standard errors (*n* 15). 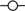, 3 % milk protein glycation (GLYC); 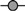, 20 % GLYC; 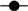, 50 % GLYC. * Significantly lower concentrations following ingestion of 50 % GLYC than 3 % GLYC (*P* < 0·001). † Significantly lower concentrations following ingestion of 20 % GLYC than 3 % GLYC (*P* ≤ 0·029). ‡ Significantly lower concentrations following ingestion of 50 % GLYC than 20 % GLYC (*P* < 0·001).

**Fig. 5.**
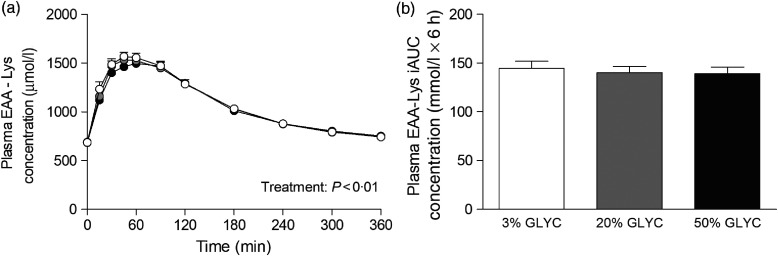
Plasma essential amino acids without lysine (EAA-Lys). Values are means with their standard errors (*n* 15). 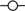, 3 % milk protein glycation (GLYC); 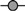, 20 % GLYC; 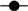, 50 % GLYC. iAUC, incremental AUC. No significant differences were observed between the milk proteins over time (a) or during the complete 6-h post-prandial period (b).

Plasma lysine concentrations are presented in Fig. 4. Plasma lysine concentrations showed a sustained increase above baseline values following ingestion of the milk proteins, with peak lysine concentrations being reached within the first 45 min after protein ingestion. The increase in plasma lysine concentrations was strongly attenuated following ingestion of the 20 and 50 % GLYC milk proteins when compared with 3 % GLYC (time × treatment effect: *P* < 0·001). The 20 % GLYC milk proteins showed lower lysine concentrations than 3 % GLYC from *t* = 30 until *t* = 240 min, and the 50 % GLYC showed lower lysine concentrations than 3 % GLYC from *t* = 15 until *t* = 360 min (*P* < 0·01; Fig. 4(a)). In accordance, the integrated plasma lysine responses were significantly different between treatments (*P* < 0·001), with the iAUC following ingestion of the 20 and 50 % GLYC being 35 (SEM 4) % and 92 (SEM 2) % lower when compared with the 3 % GLYC (21·3 (SEM 1·4) and 2·8 (SEM 0·7) *v*. 33·3 (SEM 1·7) mmol/l × 6 h, respectively; *P* < 0·001; Fig. 4(b)). Plasma EAA concentrations assessed without the contribution of lysine (Fig. 5(a)) showed a sustained increase over time (time effect: *P* < 0·001), with no differences being observed between treatments over time (time × treatment effect: *P* = 0·093). A treatment effect (*P* = 0·021) was observed, with 50 % GLYC showing lower plasma EAA (without lysine) concentrations than 3 % GLYC (*P* = 0·009; Fig. 5(a)). However, assessment of the full 6-h post-prandial period (iAUC without lysine) showed no differences between the 3, the 20 % and the 50 % GLYC (144 (SEM 7) *v*. 140 (SEM 6) *v*. 139 (SEM 7) mmol/l × 6 h, respectively; *P* = 0·274; Fig. 5(b)). An overview of the post-prandial responses of the individual EAA and NEAA is provided in online Supplementary Figs. S1 and S2. Subtle changes over time between the milk protein drinks were observed for the EAA isoleucine, histidine, methionine, phenylalanine, threonine and tryptophan (online Supplementary Fig. S1: time × treatment effect: *P* < 0·05) and NEAA aspartic acid and cysteine (online Supplementary Fig. S2: time × treatment effect: *P* < 0·05).

## Discussion

The present study assessed the impact of dietary protein glycation on post-prandial plasma amino acid availability. Ingestion of milk protein powder with a moderate (20 %) to high (50 %) glycation level resulted in an attenuated rise in plasma EAA concentrations when compared with the ingestion of milk protein with a low (3 %) glycation level. This attenuated post-prandial rise in plasma EAA concentrations was entirely attributed to a lower post-prandial plasma lysine availability.

While previous studies have shown distinct plasma amino acid responses following ingestion of different milk protein supplements^(^^2^^,^^4^^,^^6^^,^^9^^)^, there is currently limited insight into the impact of several industrial processing procedures on dietary protein digestion and amino acid absorption kinetics. The production of milk protein powders requires milk proteins to be exposed to heat treatment during drying procedures, resulting in protein glycation due to the Maillard reactions^(^^13^^–^^15^^)^. Consequently, glycation levels of commercially available protein products have been reported to range from 5 to 55 %^(^^24^^–^^26^^)^. It has been suggested that the level of protein glycation may largely impact protein digestion and amino acid absorption kinetics, resulting in an attenuated post-prandial rise in circulating amino acid levels^(^^31^^)^. In the present study, we assessed the plasma amino acid responses following the ingestion of milk protein powders with three different levels of protein glycation (3, 20 and 50 % glycation). We observed that ingestion of the same amount of protein with increasing levels of protein glycation attenuates the post-prandial rise in plasma EAA concentrations in a dose-dependent fashion. The post-prandial plasma EAA iAUC over the complete 6-h assessment period was 8 and 20 % lower following ingestion of the 20 and 50 % glycated milk proteins, respectively, when compared with the 3 % glycated protein. These differences were only evident for total EAA concentrations (Fig. 3(a) and (b)) and were not observed for total NEAA responses (Fig. 3(c) and (d)).

Assessment of individual post-prandial plasma EAA responses revealed that the lower total plasma EAA response was attributed to an attenuated rise in post-prandial plasma lysine concentrations (Fig. 4). In fact, without the contribution of lysine, no significant differences were observed in the post-prandial total plasma EAA concentrations (Fig. 5) or total amino acid responses following ingestion of the 3, 20 and 50 % glycated protein. Post-prandial plasma lysine concentrations were shown to be 35 and 92 % lower following ingestion of 20 and 50 % glycated milk protein, respectively, when compared with the 3 % glycated milk protein (Fig. 4). These findings are in line with a previous animal study that reported an attenuated post-prandial rise in circulating plasma lysine concentrations following ingestion of milk protein with a high glycation level^(^^31^^)^. In that study, pigs fed 50 % glycated dried skimmed milk showed a 60 % lower appearance of lysine in the portal vein when compared with the feeding of non-glycated dried skimmed milk. This attenuated response is suggested to result from the free ϵ-amino group of lysine that makes it extremely susceptible to react with reducing sugars during heat treatment and storage^(^^16^^,^^35^^)^. Several studies using *in vitro* gastrointestinal models indeed suggest that the blockage of lysine (forming a lysine-carbohydrate complex) decreases the ability of digestive enzymes to hydrolyse the dietary protein and to effectively liberate lysine^(^^27^^–^^29^^)^. The findings described in the present study support this by showing a strongly attenuated rise in plasma lysine concentrations following ingestion of milk proteins with increasing levels of protein glycation (Fig. 4).

It is currently unclear what the exact metabolic fate is of the blocked lysine present in milk proteins. While some blocked lysine is believed to be absorbed and released in the circulation, the majority will pass through the intestines without being absorbed and will either be fermented by intestinal bacteria or excreted in faeces^(^^30^^,^^36^^,^^37^^)^. In support, we were unable to detect any blocked lysine in the collected blood samples, implying that blocked lysine does not seem to pass the intestinal barrier and/or splanchnic circulation.

The post-prandial rise in plasma lysine availability following ingestion of the protein drinks with the higher glycation levels seemed to be more repressed than could be expected from a 20 or 50 % blocked lysine level (Fig. 4). The greater relative decline in post-prandial plasma lysine availability may be explained by the capacity of blocked lysine to compromise the enzymatic hydrolysis of non-glycated lysine present in dietary protein. Several amino acids in close proximity to blocked lysine have indeed been suggested to show impaired hydrolysis and subsequent absorption, resulting in greater faecal excretion of these amino acids^(^^31^^)^. Although some amino acids in the present study did show slightly lower post-prandial plasma amino acid responses (online Supplementary Figs. S1 and S2), it is unclear whether this can be attributed to impairments in enzymatic hydrolysis of these amino acids due to protein glycation.

The findings described in the present study show that glycation as a result of heat treatment can significantly reduce the post-prandial availability of plasma lysine. Factors such as protein glycation due to industrial processing may, therefore, need to be taken into account when assessing the nutritional quality of a protein source. In the case of milk-based products, reducing sugars (i.e. lactose) can readily react with protein lysine residues during heat treatment and during shelf storage^(^^24^^–^^26^^)^. Some reports suggest that carbohydrate-rich milk products such as skimmed milk powder, infant formula and milk-based sport supplements may show protein glycation levels as high as 55 %^(^^24^^–^^26^^)^. Consequently, while these high-quality protein sources are considered to have a more optimal amino acid profile to stimulate post-prandial muscle protein synthesis^(^^38^^,^^39^^)^, their anabolic properties may be compromised by the reduced lysine availability due to protein glycation. In support, it has been shown that reduced plasma lysine availability as seen in the present study can result in an attenuated utilisation of other protein derived (essential) amino acids^(^^40^^–^^42^^)^. For example, suboptimal amounts of ingested or infused lysine have been shown to increase oxidation of EAA, using the indicator amino acid oxidation approach^(^^41^^)^. A reduced (bio)availability of lysine due to protein glycation may, therefore, lower the actual quality of a protein source to stimulate (muscle) protein synthesis and, as such, rendering the protein source less supportive for post-prandial (muscle) growth. More research will be required to quantify the impact of protein glycation on post-prandial protein handling and subsequent muscle protein synthesis rates in humans.

In conclusion, ingestion of dietary protein with a high glycation level (20–50 %) strongly reduces the post-prandial plasma lysine availability *in vivo* in humans in a dose-dependent fashion. Consequently, protein glycation can reduce the quality of a protein and may negatively impact the anabolic properties of a protein source. This underlines the importance of taking processing induced protein modifications into account when assessing the nutritional quality of a protein source.

## Supplementary material

For supplementary material accompanying this paper visit https://doi.org/10.1017/S0007114519002927. click here to view supplementary material
