# Supplementary figures and images for: The glycation level of milk protein strongly modulates post-prandial lysine availability in humans
Source: Br J Nutr. 2019 Nov 15;123(5):545–52. doi: 10.1017/S0007114519002927 (PMC7015880; doi:10.1017/S0007114519002927)

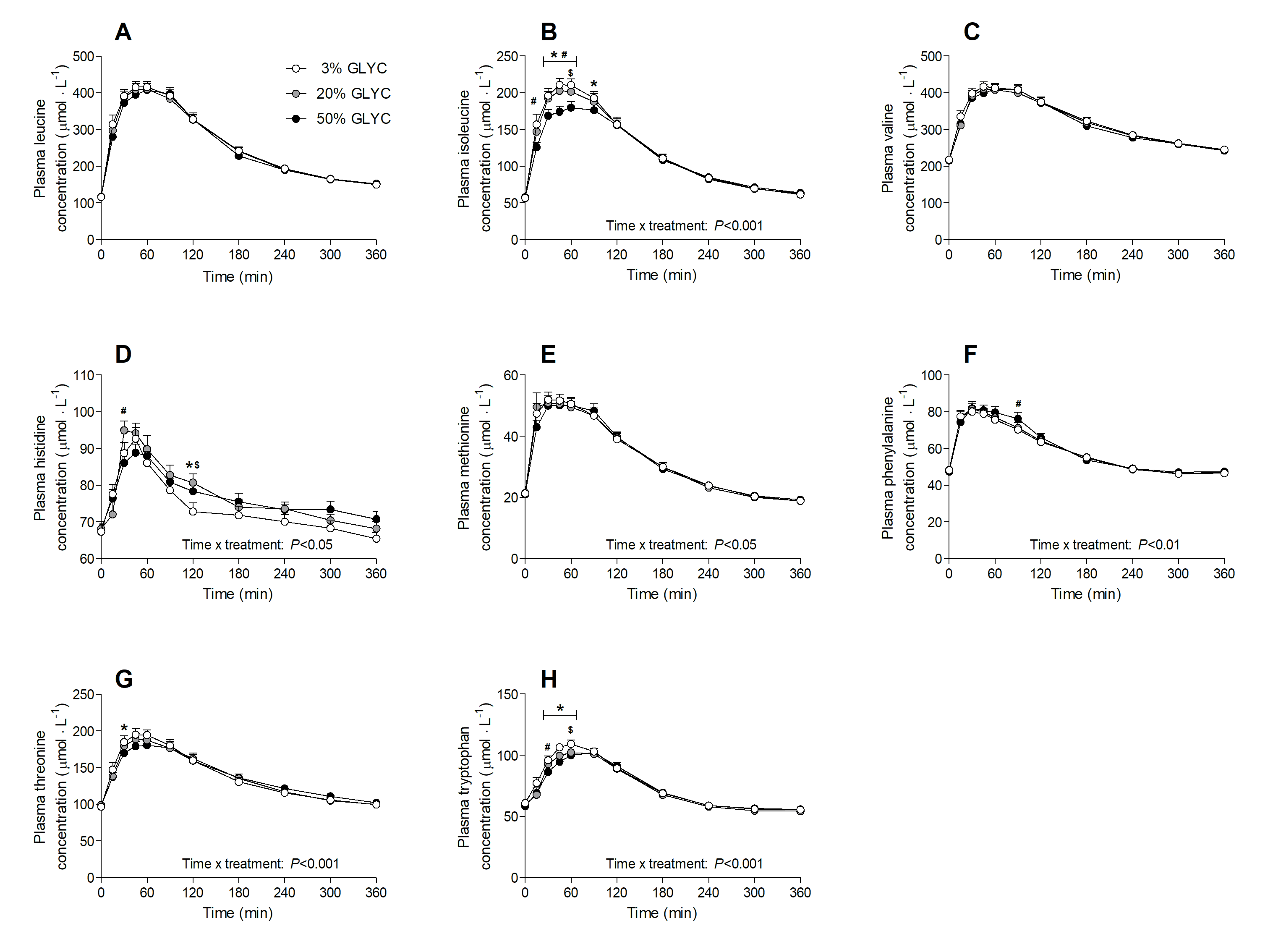

Supplement: Supplementary file 1 [file S0007114519002927sup.zip › S0007114519002927sup001.tif]

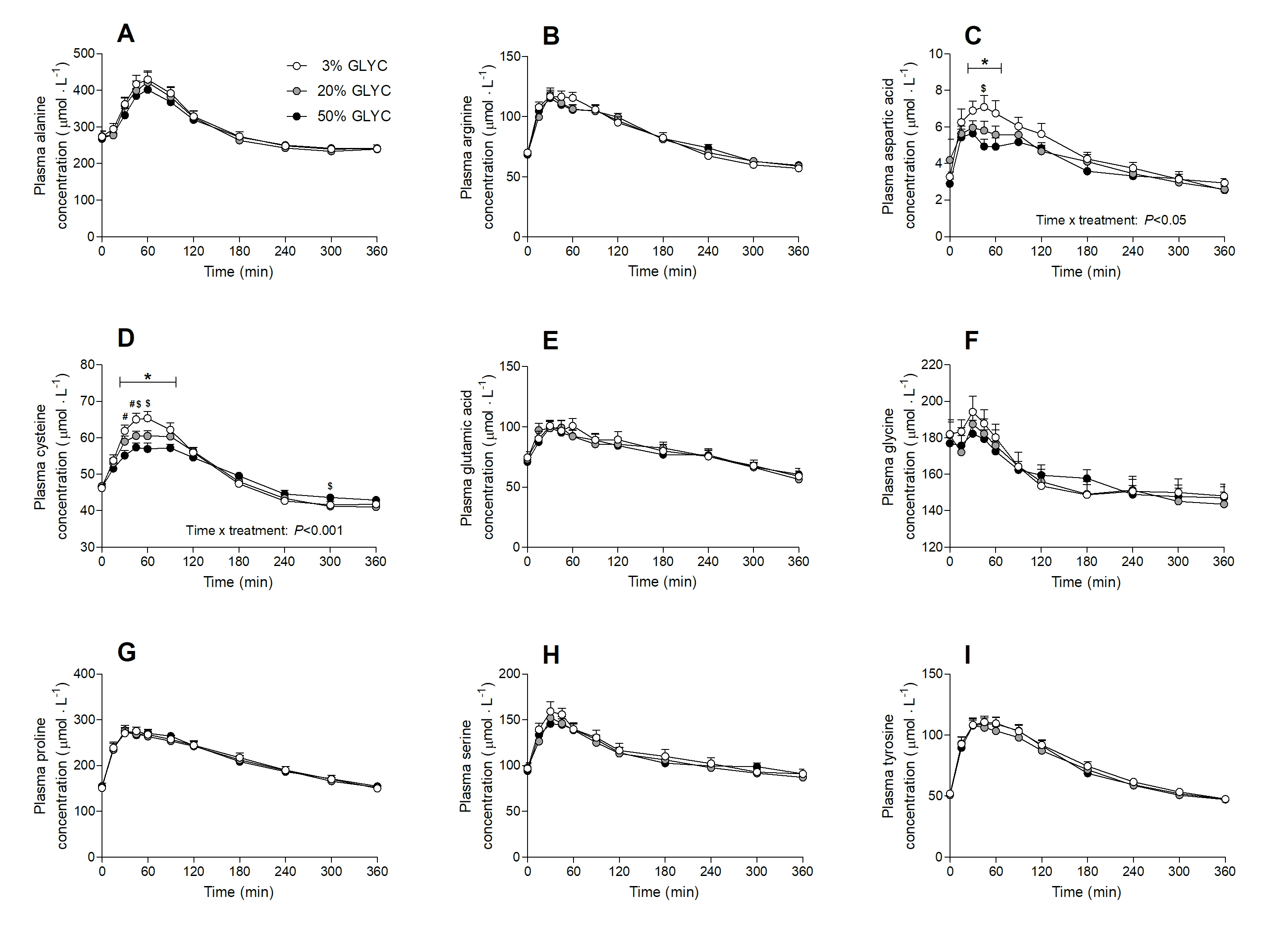

Supplement: Supplementary file 1 [file S0007114519002927sup.zip › S0007114519002927sup002.tif]
